# Protocol for generating megakaryocytes from patient induced pluripotent stem cells for disease modeling and compound screening

**DOI:** 10.1016/j.xpro.2026.104393

**Published:** 2026-02-27

**Authors:** Payal Chawla, Niclas Flosdorf, Marcelo A.S. de Toledo, Martin Zenke

**Affiliations:** 1 Department of Medicine IV, Hematology, Oncology and Stem Cell Transplantation, Faculty of Medicine, RWTH Aachen University Hospital, 52074 Aachen, Germany; 2 Center for Integrated Oncology Aachen Bonn Cologne Düsseldorf (CIO ABCD), 52074 Aachen, Germany; 3 Institute for Cell and Tumor Biology, RWTH Aachen University Medical School, 52074 Aachen, Germany

**Keywords:** Cell Biology, Cell culture, Cell-based Assays, Flow Cytometry, Stem Cells, Cell Differentiation

## Abstract

Here, we present a protocol for generating megakaryocytes from patient induced pluripotent stem cells (iPSCs). We describe steps for expanding and dissociating iPSCs into single cells and aggregating them into spin embryoid bodies (EBs). We detail procedures for lineage commitment and differentiation toward megakaryocytes within 14 days. This protocol supports phenotypic characterization and compound testing across multiple patient-derived iPSC lines.

For complete details on the use and execution of this protocol, please refer to Flosdorf et al.^1^

## Highlights

• Instructions for differentiating patient iPSCs into megakaryocytes
• Steps for efficient mesoderm induction and cytokine- and compound-guided differentiation
• Procedures for disease modeling and compound screening

## Abstract (editor highlights)

Publisher’s note: Undertaking any experimental protocol requires adherence to local institutional guidelines for laboratory safety and ethics.

## Graphical abstract

**Figure:**
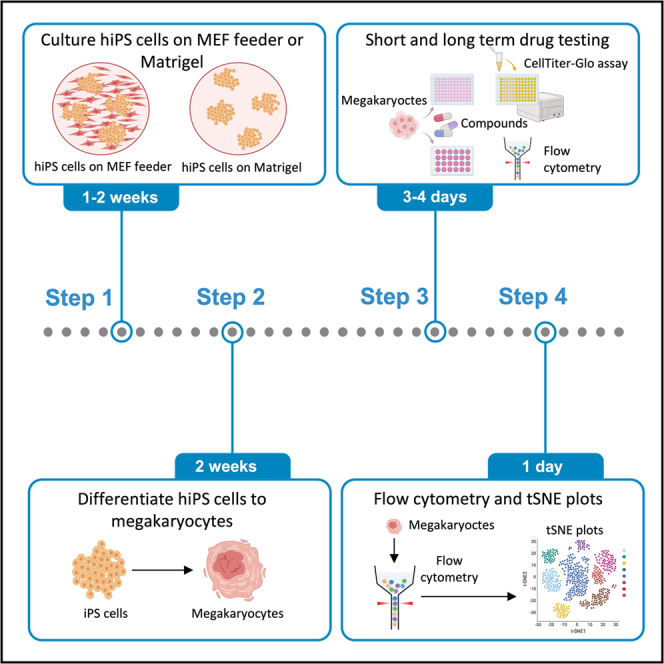

## Before you begin

Prior to starting this protocol, it is important to establish and maintain high quality human induced pluripotent stem cells (hiPS cells) using either feeder-free or feeder-based conditions. Preparation of all reagents, medias, coated culture surfaces should be carried out in advance under sterile conditions. hiPS cells used for this protocol should be stable in growth characteristics and at low-passage numbers to ensure efficient embryoid body (EB) formation and further differentiation.

### Innovation

Patient hiPS cells present themselves with a particularly high heterogeneity due to disease and patient specific features. These include disease and/or associated mutations and the patient’s genomic profile. Therefore, patient hiPS cell differentiation towards megakaryocytes has remained challenging despite a number of existing megakaryocyte differentiation protocols.^1^^,^^2^^,^^3^

To meet these challenges, we optimized critical steps of hiPS cell differentiation and assembled a workflow to achieve robust hematopoietic cell commitment and megakaryocyte differentiation within 14 days. Our protocol is compatible for both feeder-free conditions and mouse embryonic fibroblast (MEF) feeder co-culture for maintenance of hiPS cells. We use CEPT cocktail to enhance cell survival and 3D EB core formation.^4^ We incorporated the Glycogensynthase-Kinase GSK-3α/β inhibitor CHIR99021^5^ to activate Wnt signaling and to ensure efficient mesoderm commitment.

Patient and disease specific hiPS cells are an appealing cell source for disease modelling and compound screening. Accordingly, we included a section in our protocol assessing megakaryocyte phenotype in disease, analogous to our previous work on CALR and JAK2 V617F hematopoietic malignancies.^1^^,^^3^ We describe a screening platform based on the CellTiter-Glo® Luminescence assay to determine the impact of targeted compounds on differentiation, maturation and cell viability of megakaryocytes. We apply flow cytometry and t-distributed Stochastic Neighbor Embedding (tSNE) plots for megakaryocyte phenotyping.

### Institutional permissions

MEF were prepared from mouse embryos (E 13.5) using standard procedures and followed the local institutional guidelines, permit number 81-02.04.2020.A139. The patient hiPS cells are described in Olschok et al., 2021 and Flosdorf et al., 2024^1^^,^^3^ and were generated upon approval of the Ethics Committee of the Medical Faculty, RWTH Aachen University, Aachen, Germany, permit number EK206/09.

#### Preparation for hiPS cell culture on MEF feeder and Matrigel

**Timing: 0.5 day**

1. Prepare Gelatin coated plates.
  a. To prepare a 0.1% gelatin solution add 0.5 g gelatin to 500 mL distilled water.
  b. Autoclave at 121°C for 30 minutes to solubilize and sterilize gelatin.
    ***Note:*** Gelatin will solubilize during autoclaving. Store at room temperature (∼18°C) for 1–2 months or for long term storage at 4°C for 6–8 months.
  c. For coating procedure, cover surface of 6-well tissue culture dish with 1 mL gelatin solution/well and incubate at 37°C for at least 15 min.
  d. Aspirate gelatin solution prior to plating cells.
    ***Note:*** Use immediately to avoid drying of the coated surface.
2. Prepare MEF coated plates.
  a. Pre-warm MEF medium (Materials and equipment) to 37°C in the water bath.
  b. Add 10 mL MEF medium to a 15 mL conical Falcon tube.
  c. Thaw MEF vial from liquid nitrogen in 37°C water bath until the last ice crystal.
  d. Transfer MEF from the vial into the Falcon tube containing MEF medium.
  e. Centrifuge for 4 minutes at 400 g. Discard the supernatant and resuspend the cell pellet in 6 mL MEF medium as per requirement (see Note).
  f. Use the prepared gelatin coated plates to seed MEF. Remove gelatin solution from plates and add 2 mL MEF medium/well. To each well of the 6-well plate add 0.5 mL of MEF suspension. The 6 mL MEF suspension is enough for two 6-well plates.
    ***Note:*** MEF are inactivated by gamma-radiation (30 Gy; GSR C1 Blood Irradiation Device, Gamma Service Medical, Leipzig, Germany) and should be seeded with a density of 25–30,000 cells/cm^2^ on gelatin coated dishes. MEF are frozen in aliquots with 3.6×10^6^ cells per vial.
    **CRITICAL:** The quality of MEF is essential to ensure that hiPS cells are kept in an undifferentiated state during culture. Therefore, it is important to grow MEF in the above-mentioned seeding density. Overgrown MEF may cause reduction in hiPS cell adherence upon splitting.
  g. Distribute seeded MEF on the plate by gentle rocking motion.
  h. Incubate at 37°C in 5% CO_2_ incubator.
3. Prepare Matrigel coated plates.
  a. Thaw one aliquot of Matrigel at 4°C in the fridge.
  b. Dilute the aliquot in 25 mL cold KO-DMEM and mix with precooled pipette.
  c. Distribute diluted Matrigel to each well of precooled 6-well plates (1 mL/well).
  d. Seal coated plates with Parafilm and store at 4°C.
  e. Coated plates are stable in the fridge for up to 2 weeks.

***Note:*** Human embryonic stem cell (ES cell)-qualified Matrigel matrix should be aliquoted depending on the protein concentration given by the manufacturer on the certificate of analysis with a volume of 200–350 μL. Aliquots are stable for 6 months if stored at −20°C.

**CRITICAL:** Plates, pipettes and tubes should be chilled at −20°C prior to coating. This process prevents premature gelling and ensures even Matrigel coating throughout all wells.

#### Culture and expansion of hiPS cells on MEF feeder or Matrigel (step 1)

**Timing: 1-2 weeks**

**Figure 1:**
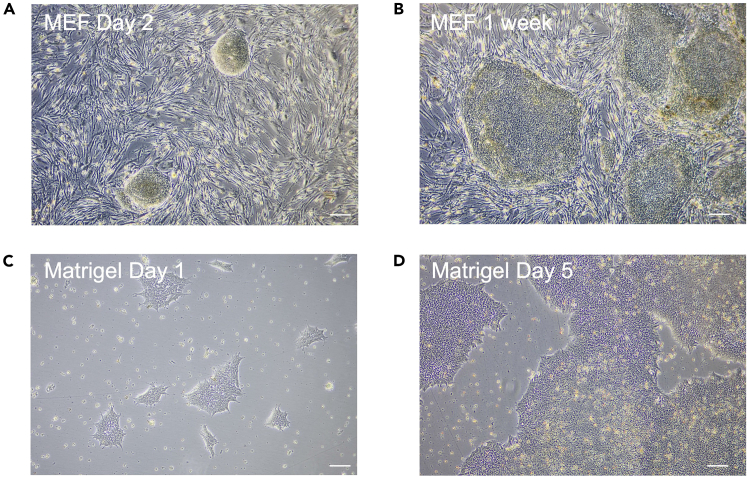
hiPS cell cuture on MEF feeder and Matrigel coated plates (A) MEF-hiPS cell co-culture at Day 2 after thawing. (B) MEF-hiPS cell co-culture upon 1 week. (C) hiPS cells on Matrigel coated plates at Day 1 after thawing. (D) hiPS cells on Matrigel coated plates on Day 5. Scale bar depicts 400 μm.

***Note:*** CEPT cocktail is used in the first passage after thawing hiPS cells but is not needed during passaging.

4. Prepare CEPT cocktail.
  a. Dissolve Chroman 1, Emricasan and trans-ISRIB each in 500 μL DMSO as stock solutions (10,000x) following the manufacturer instructions.
  b. Pipet 200 μL of dissolved Chroman 1 (0.5 mM), Emricasan (50 mM) and trans-ISRIB (7 mM) each and mix thoroughly in a 1.5 mL Eppendorf tube. Aliquot in 10 μL volumes and store at −80°C.
    ***Note:*** trans-ISRIB may not dissolve well. Always check for undissolved powder by visual inspection. Heat 5 min at 60°C and resuspend by vortexing in case of insolubility.
  c. Dissolve Polyamine supplement in 5 mL distilled water (1,000x).
  d. Filter sterile through 0.22 μm.
  e. Prepare 20 μL aliquots and store at −80°C.
  f. Add Chroman, Emricasan and trans-ISRIB mix (3,300x) as a 1 μL/3 mL concentration. Add Polyamine (1,000x) as a 1 μL/mL concentration.
5. Thaw hiPS cells on MEF coated plates (Figures 1A and 1B).
  a. Pre-warm hiPS medium (Materials and equipment).
  b. Add 10 mL hiPS medium to a 15 mL conical Falcon tube.
  c. Thaw hiPS cell vial from liquid nitrogen in 37°C water bath until the last ice crystal.
  d. Gently add hiPS cells to the Falcon tube containing hiPS medium.
  e. Centrifuge for 4 minutes at 400 g. Discard the supernatant and resuspend the cell pellet in hiPS medium containing CEPT cocktail as per requirement for the number of wells of 6-well plate.
  f. Aspirate MEF medium from MEF coated plates and wash each well with PBS. Directly discard PBS and add hiPS medium containing CEPT cocktail to each well (2 mL/well in 6-well plate).
  g. Distribute hiPS cells equally to MEF coated plate (500 μL/well) by gentle rocking motion.
  h. Incubate hiPS cells in hiPS medium at 37°C in 5% CO_2_ incubator.

6. Thaw hiPS cell lines on Matrigel coated plates (Figures 1C and 1D).
  a. Pre-warm iPS-Brew and KO-DMEM (Materials and equipment) to room temperature (∼18°C).
  b. Pre-warm Matrigel coated plate in the incubator at 37°C for Matrigel gelation.
  c. Add 10 mL KO-DMEM to a 15 mL Falcon tube.
  d. Thaw hiPS cells from liquid nitrogen in 37°C water bath until the last ice crystal.
  e. Add hiPS cells to medium tube gently.
  f. Centrifuge for 4 minutes at 400 g. Discard supernatant and resuspend cell pellet in 3 mL iPS-Brew medium containing CEPT cocktail.
  g. Aspirate Matrigel solution from the pre-coated 6-well plates, add 1 mL iPS-Brew supplemented with CEPT cocktail to each well of the 6-well plate.
  h. Distribute hiPS cells equally to the plate (500 μL/well) by gently rocking motion.
  i. Incubate hiPS cells in iPS-Brew at 37°C in 5% CO_2_ incubator.
7. Routine passage of hiPS cells on MEF coated plates.
  a. Pre-warm hiPS medium and Collagenase IV (1 mg/mL) working solution.
  b. Aspirate medium and add 1 mL Collagenase IV to each well of the 6-well plate containing hiPS cells.
  c. Incubate at 37°C in 5% CO_2_ incubator for 20–60 min.
    **CRITICAL:** Activity of Collagenase IV is strongly dependent on storage conditions. Collagenase that was stored at 4°C for over a week or underwent repeated freeze-thaw cycles performs worse than a freshly thawed Collagenase IV. Progress of colony detachment should be observed by microscopy. Stop the Collagenase treatment when edges of colonies begin to roll up.
  d. Add 2 mL KO-DMEM per well and rinse gently with a 10 mL pipette.
  e. Collect cell clumps into a 15 mL conical Falcon tube.
  f. Allow the clumps to sediment for 2–3 minutes and aspirate the medium.
    ***Note:*** Sedimentation by gravity selects for larger clusters and allows to remove small clusters and single cells. Alternatively, centrifuge for 4 min at 400 g, discard supernatant and resuspend cell pellet in 2 mL hiPS medium. After reaching a confluency of 70–80%, hiPS cells are passaged at 1:4 or 1:6 ratio. When resuspending hiPS cells avoid generating air bubbles.
  g. Aspirate MEF medium from MEF coated plates and wash each well with PBS. Directly discard PBS and add the required hiPS medium to each well (1.5 mL/well).
  h. Distribute hiPS cells equally to MEF coated plate (500 μL/well) by gentle rocking motion.
  i. Incubate at 37°C in 5% CO_2_ incubator.
    ***Note:*** For medium change, pre-warm hiPS medium in a water bath. Discard old hiPS medium. Add fresh hiPS medium (2 mL/well of 6-well plate; more medium for more dense wells). Medium change should be performed daily.
8. Routine passage of hiPS cells on Matrigel coated plates with Accutase.
  a. Pre-warm iPS-Brew, Accutase and KO-DMEM in a water bath.
  b. Pre-warm Matrigel coated plate in the incubator at 37°C for Matrigel gelation.
  c. Aspirate and add Accutase to each well (1 mL/well, 6-well plate).
  d. Incubate 37°C for 4 min.
    ***Note:*** Progress of cell detachment should be observed by microscopy.
  e. Add 2 mL KO-DMEM per well and rinse gently with 1 mL pipette.
  f. Collect cells in 15 mL conical Falcon tube containing 4 mL of KO-DMEM.
  g. Centrifuge for 4 minutes at 400 g, discard supernatant and resuspend pellet in 0.5 mL of KO-DMEM supplemented with CEPT cocktail.
  h. Perform cell counting and transfer desired number of cells to a new Matrigel coated plate already containing iPS-Brew with CEPT cocktail (1.5 mL/well). Distribute hiPS cells equally by gentle rocking motion.
  i. Incubate cells at 37°C in 5% CO_2_ incubator.
    ***Note:*** Recommended cell concentration: 1×10^5^ cells/well.
9. Routine passage of hiPS cells on Matrigel coated plates with 0.5 mM EDTA (“Clump splitting”).
  a. Pre-warm Matrigel coated plate in the incubator at 37°C for Matrigel gelation.
  b. Aspirate iPS-Brew from wells and wash twice with 0.5 mM EDTA in PBS (1 mL/well of 6-well plate) and aspirate directly.
  c. Add 1 mL 0.5 mM EDTA in PBS on well and incubate 2 minutes at room temperature (∼18°C).
  d. Aspirate the Matrigel in the meantime. Add 2 mL/well of iPS-Brew to the new Matrigel coated 6-well plate.
  e. Aspirate the 0.5 mM EDTA PBS solution from the well.
  f. Add 2 mL iPS-Brew per well using a 10 mL pipette directly on top of the cells. Resuspend only 3 times. Based on the desired cell seeding density take the volumes listed below and add to the new 6-well plate.
    ***Note:*** The colonies should come apart upon EDTA treatment but not form a single cell population.
    1:3 splitting: Take 660 μL.
    1:4 splitting: Take 500 μL.
    1:5 splitting: Take 400 μL.
    1:6 splitting: Take 334 μL.
  g. Distribute hiPS cells by gentle rocking motion to ensure the cells have dispersed around the well equally.
  h. Incubate cells at 37°C in 5% CO_2_ incubator.
    ***Note:*** Cells should form large colonies and be 85% confluent in 3–5 days (depending on the cell seeding density) for the next passaging cycle as shown in the Figure 1.
10. Freezing of hiPS cells.
  a. Pre-warm hiPS medium and Accutase to 37°C in a water bath.
  b. Pre-cool cryopreservation medium (Materials and equipment).
  c. Aspirate the medium and add Accutase to each well (1 mL/well, 6-well plate).
  d. Incubate at 37°C for 4 minutes.
  e. Add 2 mL KO-DMEM per well and rinse gently with a 10 mL pipette.
  f. Collect cells in a 15 mL conical Falcon tube.
  g. Centrifuge for 4 minutes at 400 g.
  h. Add CEPT cocktail to the cryopreservation medium.
  i. Discard supernatant and resuspend cell pellet gently in cryopreservation medium.
  j. Distribute suspension into cryovials (1 mL/vial).
  k. Place vials into −80°C freezer in Mr. Frosty or Styrofoam box to cool down slowly.
  l. Transfer frozen vials to liquid nitrogen 24 hours later for long term storage.

## Key resources table

**Table:** 

REAGENT or RESOURCE	SOURCE	IDENTIFIER
**Antibodies**		

Anti-human CD34 antibody (PE); 1:50	BioLegend	CAT#343505, RRID: AB_1732033
Anti-human CD41 antibody (BV-421); 1:50	BioLegend	CAT#303729, RRID: AB_2629626
Anti-human CD41/CD61 antibody (FITC); 1:50	Miltenyi Biotec	CAT#130-124-887, RRID: AB_2819707
Anti-human CD42b antibody (APC); 1:50	BioLegend	CAT#303912, RRID: AB_2113770
Anti-human CD45 antibody (APC-vio 770); 1:50	Miltenyi Biotec	CAT#130-110-773, RRID: AB_2905022
Anti-human CD61 antibody (FITC); 1:50	BioLegend	CAT#336403, RRID: AB_1227581
Anti-human CD117 antibody (FITC); 1:100	Invitrogen	CAT#11-1178-42, RRID: AB_2572472
Anti-human CD235a antibody (PE); 1:50	Miltenyi Biotec	CAT#130-120-613, RRID: AB_2801768
Anti-human CD61 microbeads	Miltenyi Biotec	CAT#130-051-101

**Chemicals, peptides, and recombinant proteins**		

Accutase	PAN	CAT#P10-21500
BMP-4	Miltenyi Biotec	CAT#130-111-165
BSA detoxified (20%)	Panzenböck et al. 1998^6^	N/A
β-Mercaptoethanol	Gibco	CAT#31350-010
CEPT cocktail	Tocris	CAT#7991
Chemically defined lipid concentrates	Gibco	CAT#11905031
CHIR99021	Tocris	CAT#4423/10
Collagenase IV	Gibco	CAT#17104-019
Distilled water (Ampurwa™)	Fresenius Kabi	CAT#PZN 10333429
DMSO	Sigma-Aldrich	CAT#D8418
DMEM, high glucose	Gibco	CAT#41965-039
DPBS (Ca^2+^/Mg^2+^ free)	Gibco	CAT#14190-094
EDTA	Gibco	CAT#15575-038
FCS	Gibco	CAT#1SB016
FGF2/bFGF	Peprotech	CAT#100-18B
Gelatin	Sigma	CAT#G1890
GlutaMAX	Gibco	CAT#35050-038
Ham’s F12 medium	Gibco	CAT#11765-054
Human albumin (Albutein)	Grifols	CAT#PZN 10408498
Human TruStain FcX™	BioLegend	CAT#422301
IMDM medium	Gibco	CAT#12440-053
StemMACS iPS-Brew XF (iPS-Brew)	Miltenyi Biotec	CAT#130-104-368
Knockout DMEM	Gibco	CAT#10829018
Knockout serum replacement	Gibco	CAT#10828028
L-Ascorbic acid	Stemcell Technologies	CAT#72132
Matrigel® hESC-Qualified Matrix	Corning	CAT#SPC-354277
Mouse embryonic fibroblast	Qin et al., 2014^7^	N/A
Non-Essential amino acids	Gibco	CAT#11140-035
Penicillin/Streptomycin 10,000 U/mL	Gibco	CAT#15140-122
Ruxolitinib	MedChem Express	CAT#HY-50856
SCF	Miltenyi Biotec	CAT#130-96-695
1-Thioglycerol	Sigma	CAT#M1753
Holo-Transferrin	Sigma	CAT#T0665
VEGF	Miltenyi Biotec	CAT#130-109-386

**Critical commercial assays**		

CellTiter-Glo® Luminescent Cell Viability Assay	Promega	CAT#G7571

**Experimental models: cell lines**		

hiPS cells from JAK2V617F patients	Flosdorf et al., 2024^1^	N/A

**Software and algorithms**		

FlowJo™v10.10	BD	https://www.flowjo.com/solutions/flowjo/downloads
Prism 10	GraphPad	https://www.graphpad.com/scientific-software/prism/

**Other**		

6-well tissue culture plate	Greiner	CAT#657160
96-well U-bottom suspension microtiter plate	Greiner	CAT#650185
Reagent reservoir for multichannel pipette (60 mL)	Brand	CAT#703459
15 mL Falcon tubes	Corning	CAT#352096
50 mL Falcon tubes	Corning	CAT#352070
FACS tubes	Sarstedt	CAT#55.1579
Media bottle, 250 mL	Starlab	CAT#CC8221-4000
Bottle top filtration unit, 0.22 μm	Starlab	CAT#CC8222-4226
Cell strainer 70 μm	Corning	CAT#352350
FACS Canto II	BD Biosciences	FACS Canto II website
Infinite 200 PRO	TECAN	TECAN website

## Materials and equipment

***Note:*** All Reagents, media and cytokine solutions are prepared under sterile conditions under laminar flow hood.

### MEF culture medium

To formulate MEF culture medium for MEF cell culture, combine the following reagents: 220 mL High-glucose DMEM (DMEM), 25 mL FCS (10%), 2.5 mL Penicillin-Streptomycin (1%) and 2.5 mL GlutaMAX (2 mM). Mix thoroughly in a sterile 250 mL media bottle.

**Table:** 

Reagent	Final concentration	Amount
High-glucose DMEM	–	220 mL
FCS	10%	25 mL
Penicillin/Streptomycin	1%	2.5 mL
GlutaMAX	2 mM	2.5 mL
**Total**	N/A	**250 mL**

Store at 4°C for up to 2 weeks.

### hiPS culture medium

To formulate hiPS culture medium for iPS cell culture, combine the following reagents: 192 mL Knockout DMEM (DMEM), 50 mL Knockout serum replacement (20%), 2.5 mL Non-Essential amino acid solution (0.1 mM), 2.5 mL Penicillin-Streptomycin (1%) and 2.5 mL GlutaMAX (2 mM), 500 μL β-Mercaptoethanol (0.1 mM) and 25 μL bFGF (10 ng/mL). Mix thoroughly in a sterile 250 mL media bottle.

**Table:** 

Reagent	Final concentration	Amount
Knockout DMEM	–	192 mL
Knockout serum replacement	20%	50 mL
Non-Essential amino acid	0.1 mM	2.5 mL
Penicillin/Streptomycin	1%	2.5 mL
GlutaMAX	2 mM	2.5 mL
β-Mercaptoethanol	0.1 mM	500 μL
bFGF (stock solution 100 μg/mL)	10 ng/mL	25 μL
**Total**	N/A	**250 mL**

Store at 4°C for up to 2 weeks.

### iPS-Brew culture medium

To formulate iPS-Brew culture medium for iPS cell culture, combine 500 mL of human StemMACS PSC-Brew Basal Medium XF with 10 mL of human StemMACS PSC-Brew Supplement XF (50x) directly in the medium XF bottle. Store at 4°C.

### Cryopreservation medium

To formulate the cryopreservation medium, combine 45 mL FCS (90%) with 5 mL DMSO (10%) in a 50 mL Falcon tube. Always prepare fresh and keep on ice.

### Collagenase IV

To prepare this reagent dissolve 1 g of Collagenase IV (1 mg/mL) in 1000 mL Knockout MEM solution. Sterile filter over a 0.22 μm bottle top filtration unit. Aliquot and keep at 4°C for short term or −20°C for long-term storage.

### 0.5 mM EDTA

To prepare this reagent dissolve 50 μL of 0.5 M EDTA (0.5 mM) into 50 mL of DPBS (Ca^2+^/Mg^2+^ free) in a 50 mL Falcon tube. This reagent can also be stored at room temperature (∼18°C) for 1 month.

### Basic FGF/bFGF

To prepare this reagent dissolve 50 μg bFGF (100 μg/mL) and 5 μL Bovine serum albumin 10% (0.1%) in 500 μL of DPBS (Ca^2+^/Mg^2+^ free) solution.

**Table:** 

Reagent	Final concentration	Amount
bFGF	100 μg/mL	50 μg
Bovine Serum Albumin 10%	0.1%	5 μL
DPBS (Ca^2+^/Mg^2+^ free)	N/A	**500 μL**

Aliquot e.g., 25 μL/vial or 100 μL/vial and store at −20°C for up to 6 months.

### Bone morphogenetic factor 4/BMP-4

To prepare this reagent dissolve 25 μg of BMP-4 (25 μg/mL) and 10 μL of Bovine Serum Albumin 10% in 1 mL of DPBS (Ca^2+^/Mg^2+^ free) solution.

**Table:** 

Reagent	Final concentration	Amount
BMP-4	25 μg/mL	25 μg
Bovine Serum Albumin 10%	0.1%	10 μL
DPBS (Ca^2+^/Mg^2+^ free)	N/A	**1 mL**

Aliquot e.g., 20 μL/vial and store at −20°C for up to 6 months.

### Stem cell factor/SCF

To prepare this reagent dissolve 25 μg of SCF (100 μg/mL) and 2.5 μL of Bovine Serum Albumin 10% (0.1%) in 250 μL of DPBS (Ca^2+^/Mg^2+^ free) solution.

**Table:** 

Reagent	Final concentration	Amount
SCF	100 μg/mL	25 μg
Bovine Serum Albumin 10%	0.1%	2.5 μL
DPBS (Ca^2+^/Mg^2+^ free)	N/A	**250 μL**

Aliquot e.g., 20 μL/vial and store at −20°C for up to 6 months.

### Thrombopoietin/TPO

To prepare this reagent dissolve 50 μg of TPO (20 μg/mL) and 25 μL of Bovine Serum Albumin 10% (0.1%) in 2.5 mL of DPBS (Ca^2+^/Mg^2+^ free) solution.

**Table:** 

Reagent	Final concentration	Amount
TPO	20 μg/mL	50 μg
Bovine Serum Albumin 10%	0.1%	25 μL
DPBS (Ca^2+^/Mg^2+^ free)	N/A	**2.5 mL**

Aliquot e.g., 50 μL/vial and store at −20°C for up to 6 months.

### Vascular endothelial growth factor/VEGF

To prepare this reagent dissolve 100 μg of VEGF (25 μg/mL) and 40 μL of Bovine Serum Albumin 10% (0.1%) in 4 mL of DPBS (Ca^2+^/Mg^2+^ free) solution.

**Table:** 

Reagent	Final concentration	Amount
VEGF	25 μg/mL	100 μg
Bovine Serum Albumin 10%	0.1%	40 μL
DPBS (Ca^2+^/Mg^2+^ free)	N/A	**4 mL**

Aliquot e.g., 20 μL/vial and store at −20°C for up to 6 months.

### CHIR99021

To prepare this reagent dissolve 2 mg of CHIR99021 (10 mM) in 429.8 μL of DMSO. Warm to 37°C to facilitate solubilization. Aliquot e.g., 20 μL/vial and store at −20°C for up to 6 months.

### L-ascorbic acid/LAA

To prepare this reagent dissolve 500 mg of LAA (50 mg/mL) in 10 mL distilled water. Sterile filter over 0.22 μm filter. Aliquot e.g., 250 μL/vial and store at −20°C for up to 1 year. Protect from light. Always add fresh to the medium.

### Monothioglycerol/MTG

To prepare this reagent dissolve 87 μL of MTG (100 mM) in 10 mL distilled water. MTG has a high viscosity, pipette slowly in order to dispense accurately. Aliquot e.g., 1 mL/vial and store at −20°C for up to 1 year. Always add fresh to the medium.

### Holo-Transferrin

To prepare this reagent dissolve 500 mg of Holo-Transferrin (17.5 mg/mL) in 28.55 mL of distilled water. Rotate at 4°C for 1 hour. Sterile filter over 0.22 μm filter. Aliquot e.g., 1 mL/vial and store at −20°C for up to 1 year. Always add freshly to the medium.

### Day 0 serum-free basal medium

To formulate the Day 0 serum-free medium (SFM) basal medium for Spin EB culture combine 9.5 mL IMDM (50 %), 9.5 mL HAM’s F-12 (50 %), 0.5 mL BSA detoxified (0.5%), 0.2 mL chemically defined lipid concentrate (1%), 0.2 mL GlutaMAX (2mM) and 80 μL MTG (400 μM) in a 50 mL Falcon tube.

**Table:** 

Reagent	Final concentration	Amount
IMDM	50 %	9.5 mL
HAM’s F-12	50 %	9.5 mL
BSA detoxified	0.5 %	0.5 mL
Chemical defined lipid concentrate	1%	0.2 mL
GlutaMAX	2 mM	0.2 mL
MTG	400 μM	80 μL
**Total**	N/A	**20 mL**

Sterile filter over 0.22 μm filter. Store at 4°C for up to 2 weeks.

***Note:*** BSA is used only for EB formation during Day 0. 20 mL SFM are used for four 96-well plates of Spin EB culture.

Shortly before use, prepare the Day 0 Spin EB medium using the Day 0 SFM basal medium. In 20 mL SFM basal medium add the following reagents: 8 μL BMP-4 (10 ng/mL), 2 μL bFGF (10 ng/mL), 20/7 μL CEPT (1000x/3300x), 20 μL LAA (50 μg/mL) and 6.7 μL Holo-Transferrin (6 μg/mL).

**Table:** Day 0 Spin EB medium

Reagent	Final concentration	Amount
SFM	–	20 mL
BMP-4	10 ng/mL	8 μL
bFGF	10 ng/mL	2 μL
CEPT	1000x/3300x	20/7 μL
LAA	50 μg/mL	20 μL
Holo-Transferrin	6 μg/mL	6.7 μL

### Day 2 SFM basal medium

To formulate the Day 2 SFM basal medium for Spin EB culture combine 119 mL IMDM (50 %), 119 mL HAM’s F-12 (50 %), 6.25 mL Albutein (0.5%), 2.5 mL Chemically defined lipid concentrate (1%), 2.5 mL GlutaMAX (2mM) and 1 mL MTG (400 μM) in a 250 mL media bottle.

**Table:** 

Reagent	Final concentration	Amount
IMDM	50 %	119 mL
HAM’s F-12	50 %	119 mL
Albutein	0.5 %	6.25 mL
Chemical defined lipid concentrate	1%	2.5 mL
GlutaMAX	2 mM	2.5 mL
MTG	400 μM	1 mL
**Total**	N/A	**250 mL**

Sterile filter over 0.22 μm filter. Store at 4°C for up to 2 weeks.

### Day 2 spin EB medium

Shortly before use, prepare the Day 2 Spin EB medium using the Day 2 SFM basal medium. In 20 mL SFM basal medium add the following: 8 μL BMP-4 (10 ng/mL), 2 μL bFGF (10 ng/mL), 8 μL VEGF (10 ng/mL), 10 μL SCF (50 μg/mL), 20 μL CHIR 992021 (10 μM), 20 μL LAA (50 μg/mL) and 6.7 μL Holo-Transferrin (6 μg/mL).

**Table:** 

Reagent	Final concentration	Amount
SFM		20 mL
BMP-4	10 ng/mL	8 μL
bFGF	10 ng/mL	2 μL
VEGF	10 ng/mL	8 μL
SCF	50 μg/mL	10 μL
CHIR99201	10 μM	20 μL
LAA	50 μg/mL	20 μL
Holo-Transferrin	6 μg/mL	6.7 μL

### Day 3–8 spin EB medium

Shortly before use, prepare the Day 3–8 Spin EB medium using the Day 2 SFM basal medium. In 20 mL SFM basal medium add the following: 8 μL BMP-4 (10 ng/mL), 2 μL bFGF (10 ng/mL), 8 μL VEGF (10 ng/mL), 10 μL SCF (50 μg/mL), 20 μL LAA (50 μg/mL) and 6.7 μL Holo-Transferrin (6 μg/mL).

**Table:** 

Reagent	Final concentration	Amount
SFM		20 mL
BMP-4	10 ng/mL	8 μL
bFGF	10 ng/mL	2 μL
VEGF	10 ng/mL	8 μL
SCF	50 μg/mL	10 μL
LAA	50 μg/mL	20 μL
Holo-Transferrin	6 μg/mL	6.7 μL

### Day 8–10 spin EB medium

Shortly before use, prepare the Day 8–10 Spin EB medium using the Day 2 SFM basal medium. In 20 mL SFM basal medium add the following: 2 μL bFGF (10 ng/mL), 10 μL SCF (50 μg/mL), 20 μL LAA (50 μg/mL) and 6.7 μL Holo-Transferrin (6 μg/mL).

**Table:** 

Reagent	Final concentration	Amount
SFM		20 mL
bFGF	10 ng/mL	2 μL
SCF	50 μg/mL	10 μL
LAA	50 μg/mL	20 μL
Holo-Transferrin	6 μg/mL	6.7 μL

### Day 10–14 spin EB medium

Shortly before use, prepare the Day 10–14 Spin EB medium using the Day 2 SFM. basal medium. In 20 mL SFM basal medium add the following: 10 μL SCF (50 μg/mL), 20 μL TPO (20 ng/mL), 20 μL LAA (50 μg/mL) and 6.7 μL Holo-Transferrin (6 μg/mL).

**Table:** 

Reagent	Final concentration	Amount
SFM		20 mL
SCF	50 μg/mL	10 μL
TPO	20 ng/mL	20 μL
LAA	50 μg/mL	20 μL
Holo-Transferrin	6 μg/mL	6.7 μL

### FACS buffer

To prepare the FACS buffer dissolve 1.25 mL of FCS (0.5%) and 2.5 mL of EDTA (5 mM) in 246.5 mL of DPBS (Ca^2+^/Mg^2+^ free) solution in a 250 mL media bottle.

**Table:** 

Reagent	Final concentration	Amount
FCS	0.5 %	1.25 mL
EDTA	5 mM	2.5 mL
DPBS (Ca^2+^/Mg^2+^ free)	N/A	**250 mL**

Store at 4°C for up to 2 weeks.

### Antibodies for FACS staining (day 14)

Prepare a master mix for the hematopoietic progenitor cell panel by combining CD45 (APCVio770), CD34 (PE), and CD117 (FITC) antibodies as per the dilutions mentioned below.

Prepare a master mix for the megakaryocyte panel by combining CD45 (APCVio770), CD41/61 (FITC), CD42b (APC) and CD235a (PE) antibodies as per the dilutions mentioned below.

**Table:** 

Reagent	Final concentration	Clone
CD45 (dilution-1:50)	Hematopoietic progenitor cells/Megakaryocytes	REA747
CD34 (dilution-1:50)	Hematopoietic progenitor cells	581
CD117 (dilution-1:100)	Hematopoietic progenitor cells	104D2
CD41/61 (dilution-1:50)	Megakaryocytes	REA647
CD42b (dilution-1:50)	Megakaryocytes	HIP1
CD235a (dilution-1:50)	Red cells	REA175

Always prepare fresh. Keep on ice or at 4°C.

### Antibodies for FACS staining (day 18)

Prepare a master mix for the megakaryocyte panel by combining antibodies same as Day 14 as per the dilutions mentioned below.

**Table:** 

Reagent	Final concentration	Clone
CD45 (dilution-1:50)	Hematopoietic progenitor cells/Megakaryocytes	REA747
CD41/61 (dilution-1:50)	Megakaryocytes	REA647
CD42b (dilution-1:50)	Megakaryocytes	HIP1
CD235a (dilution-1:50)	Red cells	REA175

Always prepare fresh. Keep on ice or at 4°C.

## Step-by-step method details

**Timing: 1 day (for step 1)**

**Timing: 2 weeks (for step 2)**

**Timing: 0.5 day (for step 3)**

**Timing: 66–72 h (for step 4)**

**Timing: 0.5 day (for step 5)**

**Timing: >0.5 day (for step 6)**

**Timing: 0.5 day (for step 7)**

**Figure 2:**
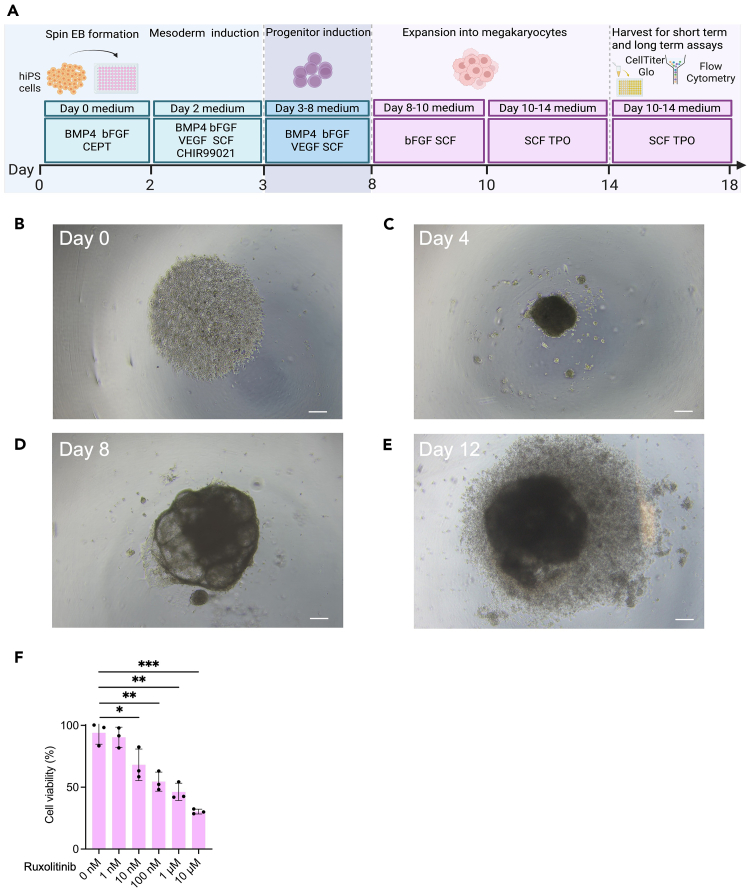
Megakaryocyte differentiation workflow (A) Differentiation scheme of hiPS cells into megakaryocytes. (B) Day 0, EB formation. (C) Day 4, EB core formation upon incubation with 1 day treatment of CHIR99201. (D) Larger EB on Day 8. (E) Progenitor halo surrounding the EB on Day 12. Scale bar depicts 400 μm. (F) CellTiter-Glo assay results on JAK2 V617F homozygous megakaryocytes (Day 18) depicting the activity of the JAK inhibitor ruxolitinib. DMSO, vehicle control. ∗ p<0.05, ∗∗ p<0.01 and ∗∗∗ p<0.001.

Spin EB differentiation of hiPS cells into hematopoietic progenitors (Step 2 and Figures 2A–2F).

1. Seeding of hiPS cells with Accutase for Spin EB formation.
  ***Note:*** After thawing, culture hiPS cells for at least 2 passages on MEF or Matrigel coated 6-well plates prior to starting the spin EB protocol.
  a. Pre-warm iPS-Brew, Accutase and KO-DMEM to 37°C.
  b. Aspirate old medium and add 1 mL Accutase to each well of a 6-well plate.
  c. Incubate at 37°C for 4 minutes.
    ***Note:*** Progress of cell detachment should be observed by microscopy.
  d. Add 2 mL KO-DMEM per well and rinse gently with a 1 mL pipette.
  e. Collect cells and strain using a 0.70 μm cell strainer in a 50 mL Falcon tube containing 5 mL of KO-DMEM.
    **CRITICAL:** This step is crucial to eliminate carry over of larger cell aggregates.
  f. Centrifuge for 4 minutes at 400 g.
  g. Discard supernatant and resuspend pellet in 0.5 mL of KO-DMEM containing CEPT cocktail.
  h. Count cells, resuspend 2 x 10^6^ cells in 5 mL KO-DMEM + CEPT cocktail and centrifuge for 4 minutes at 400 g. Discard supernatant.
    ***Note:*** The seeding density can vary between 3000–5000 cells per well of a 96-well suspension type U-bottom microtiter plate.
  i. Resuspend cells in 20 mL of Day 0 Spin EB medium and transfer to the reagent reservoir for multichannel pipette.
  j. Use a multichannel pipette to add 50 μL of cell suspension per well of a 96-well suspension type U-bottom microtiter plate.
  k. Centrifuge the U-bottom plate at 400 g for 5 minutes.
    **CRITICAL:** The acceleration and deceleration of the centrifuge should be set to low in order to allow for a smooth and gentle collection of hiPS cells in the center of the well.
  l. Use microscopy to check that the cells in wells are packed together in the center (Figure 2B, Day 0).
    ***Note:*** If cells did not form a well-packed core in the center of the U-bottom well, centrifuge again for 5 min at 400 g. After 48 h of seeding hiPS cells EB formation should be observed. EB should have a defined border with a light dense core and should grow over time (Day 4, 8 and 12, Figures 2C–2E).
2. Differentiation into hematopoietic progenitors and further into megakaryocytes.
  a. On Day 2, top up the wells with Day 2 Spin EB medium (Figure 2A).
  b. Starting on Day 3 proceed with daily partial medium change using Day 3–8 Spin EB medium.
  c. On Day 8 proceed with daily partial medium change with Day 8–10 Spin EB medium for progenitor expansion.
  d. On Day 10 proceed with daily partial medium change with Day 10–14 Spin EB medium for progenitor expansion.

***Note:*** The volume of medium removed from wells (about 75 μL), while performing daily partial medium changes, can vary due to loss of medium by evaporation in the incubator, pipetting variances etc.

**CRITICAL:** Perform partial medium change daily and add new cytokines with the fresh medium.

3. Harvest of EB, hematopoietic and megakaryocyte progenitors.
  a. Harvest EB and progenitors on Day 14 using a 1 mL pipette tip.
    **CRITICAL:** By gentle pipetting, EB and progenitors can be easily resuspended and harvested. Avoid air bubbles.
  b. Collect EB and progenitors in a 50 mL Falcon tube, centrifuge for 4 minutes at 400 g.
  c. Resuspend cells in Day 10–14 Spin EB medium.
    ***Note:*** Cell seeding density should be 1x 10^6^ cells/mL e.g. in well of a 6-well plate or 10 cm dish.
  d. After 2 days of expansion (Day 16), add 5 mL of fresh medium. Two days later (Day 18) perform a full medium change by harvesting, spinning down and resuspending cells in 10 mL of fresh Day 10–14 Spin EB medium.
  e. Continue this feeding regimen throughout the expansion phase (Day 14–18 and longer for other cell types). Monitor hematopoietic cell development/maturation and megakaryocyte markers by FACS analysis (see below 5).
    ***Note:*** Progenitors are observed morphologically by microscopy around Day 8. The longer the cells are kept in expansion phase, more mature myeloid (granulocytes, macrophages and mast cells) and less progenitor cells are obtained.
4. CellTiter-Glo® assay for short term drug testing of progenitors and megakaryocytes (Step 3).
  a. Upon cell harvest (Day 14), resuspend cells at a cell density of 115,000 cells/mL in Day 10–14 Spin EB medium (Materials and equipment) in a 50 mL Falcon tube.
    ***Note:*** 90 μL of cell suspension will contain approximately 10,000 cells. Shake the Falcon tube ever few minutes to make sure that cells are in a homogenous suspension while distributing into 96-well suspension type U-bottom microtiter plate.
  b. Prepare 5 Eppendorf tubes for compound dilution.
  c. Prepare serial dilutions as follows:
    Tube 1 – 100 μM: 495 μL solvent + 5 μL compound stock (10 mM).
    Tube 2 – 10 μM: 450 μL solvent + 50 μL of Tube 1.
    Tube 3 – 1 μM: 450 μL solvent + 50 μL of Tube 2.
    Tube 4 – 100 nM: 450 μL solvent + 50 μL of Tube 3.
    Tube 5 – 10 nM: 450 μL solvent + 50 μL of Tube 4.
    **CRITICAL:** Vortex each tube thoroughly prior to the next dilution step to dissolve the compound evenly in solvent. Keep aliquots on ice until use.
    ***Note:*** Compound aliquots are prepared and stored at −80°C at a concentration of 10 mM.
  d. Prepare the microtiter plate by distributing 10 μL of the compound in triplicates following the plate design for each concentration. Control wells are supplemented with DMSO solvent.
  e. Add 90 μL of the cell suspension into the wells of the 96-well U-bottom plate. Incubate for 66–72 hours at 37°C in 5% CO_2_ incubator.
  f. Proceed with the CellTiter-Glo® assay according to manufacturer instructions.
  g. Equilibrate the 96-well plate under the laminar flow hood for 15 minutes.
  h. Add 100 μL of CellTiter-Glo® reagent to each well and incubate for 30 minutes at room temperature (∼18°C) upon thorough mixing.
  i. Transfer the cells into an opaque 96-well flat bottom microtiter plate and measure the luminescence with a plate reader (key resources table).
  j. Analyze the results by GraphPad Prism as depicted in Figure 2F.
5. Flow cytometry analysis of progenitors and megakaryocytes (Step 4).
  a. Harvest cells, count and put up to 1 x 10^6^ cells in FACS tube, centrifuge for 5 minutes at 400 g.
  b. Resuspend in 1 mL FACS buffer, vortex and centrifuge for 5 minutes at 400 g.
  c. Discard supernatant and resuspend in 100 μL FACS buffer.
  d. Add 5 μL human TruStain FcX (Fc receptor blocking solution) and incubate for 5 minutes at room temperature (∼18°C).
  e. Prepare antibody master mix in 100 μL FACS buffer per sample as described in the Materials and equipment section for Day 14 and Day 18 (long-term drug screening).
  f. Add the antibodies and vortex.
  g. Incubate for 30 minutes at 4°C in the dark.
  h. Upon incubation add 1 mL FACS buffer to each tube, vortex, and centrifuge for 5 minutes at 400 g (Wash step 1).
  i. Discard supernatant to remove unbound antibody, repeat the washing step.
  j. Add 350 μL FACS buffer and resuspend cells by vortexing.
  k. Subject samples to flow cytometry analysis and acquire data.

**Figure 3:**
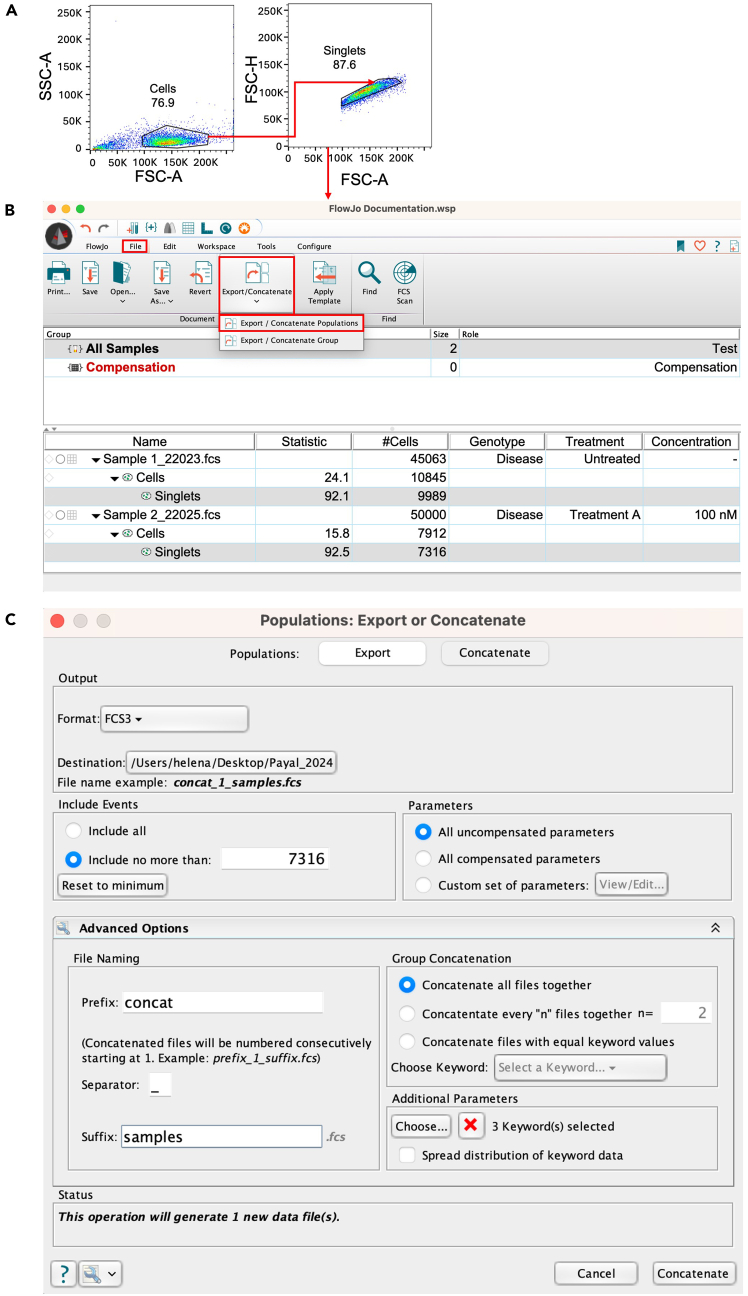
Flow cytometry gating strategy (A) Gating strategy to build the singlets gate. (B) Steps for concatenation of singlets. (C) Parameters for selection during concatenation process.

**Figure 4:**
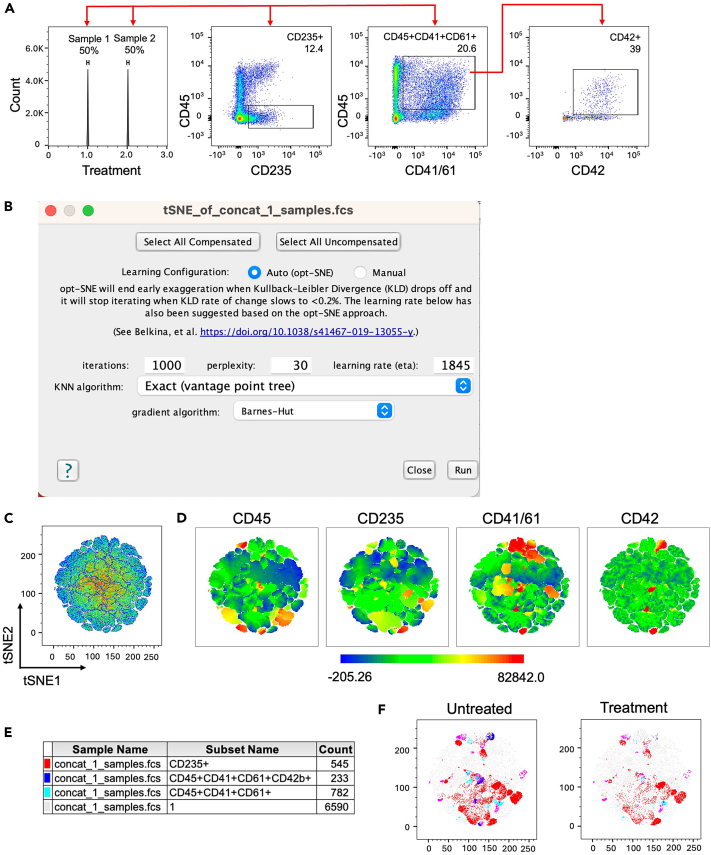
tSNE analysis of megakaryocytes (A) Gating strategy post concatenation. Segregation of samples based on treatment function, gate for red blood cells (CD45 vs CD235), megakaryocytes (CD45 vs CD41/CD61) and subsequently gated for maturation marker CD42. (B) Selection of parameters for generating a tSNE plot. (C) Example of the two samples files generated into tSNE plot. (D) Automatic overlay of markers of interest in the panel. (E) Legend for changing colors. (F) Interpretation of results for the samples.

***Note:*** Progenitor cell growth and differentiation entail culture with cytokines, such as SCF and TPO, which upon binding to cognate receptor cause receptor internalization. Consequently, such receptors, e.g. SCF receptor KIT, is not accessible for surface staining and flow cytometry analysis. Thus, resuspend the progenitor cell pellet in RPMI + 1% L-Glu + 1% P/S without cytokines (1mL RPMI/1 million cells) and transfer into an appropriate cell culture dish. This starvation step is conducted for 1.5 h at 37°, 5% CO_2._ After starvation, collect cells and centrifuge at 400 g for 5 min. Work fast, keep cells cold, and use pre-cooled solutions. This will prevent capping of antibodies on the cell surface and non-specific cell labeling. Gating strategy shown in Figures 3 and 4.

6. Visualization of flow cytometry data (Figure 3).
  a. Load the raw data files (e.g., .fcs files) into the FlowJo workspace.
  b. Label each file with additional information by clicking “Workspace”, then “Add Keyword”.
    ***Note:*** Adding more information as an indicator can be completed while acquiring data on the flow cytometry instrument of choice. However, if the samples were not labelled initially, this can be accomplished by the above information. Keywords such as “Genotype”, “Treatment” and “Concentration” can be added to the workspace as shown in Figure 3B.
  c. Create a “Cells” gate and subsequently create a “Singlets” gate (Figure 3A).
    **CRITICAL:** When using a dimensionality reduction function such as tSNE, it is important to work with a cleaner version of the raw file. Therefore, Cells and Singlets gate are imperative.
  d. Select “Singlets” from one sample. Right click and chose “Select equivalent nodes” option.
    ***Note:*** Selecting equivalent nodes allows you to automatically select the “Singlets” gate (here) across all the samples present in the workspace.
  e. Click the “File” tab and select the “Export/Concatenate” populations. (Figure 3B) A pop-up window appears. Select the following iterations as shown in Figure 3C:
    i. Choose the Populations: “Concatenate” option.
    ii. Confirm that the format is “FCS3”.
    iii. Choose the destination as appropriate.
    iv. Click on the “include no more than” option.
    v. Leave the parameters tab at “All uncompensated parameters”.
    vi. Click on “Advanced Options” and select a file name under the “Prefix” and “Suffix” tab.
    vii. Choose “Concatenate all files together” option.
    viii. Under “Additional Parameters”, choose the keywords that were inserted at the start of the workflow (Bullet point 6b).
    ix. Uncheck the option “Spread distribution of keyword data”.
    x. Click “Concatenate”.
    xi. A new pop-up window appears that allows you to choose between “New workspace” and “Existing workspace” as appropriate.
      ***Note:*** In our analysis we prefer to generate a new workspace.
    xii. A new file that has combined all the samples then appears in the workspace.
  f. Select the concatenated file and following the strategy in Figure 4A, select the Histogram option on the y-axis and plot the population against Treatment.
  g. Equal populations appear. Right click and choose “Select gates on peaks”. This function automatically labels the peaks/populations of interest and can be re-labelled as appropriate in the workspace.
    ***Note:*** Since two samples were concatenated together, they are depicted as two cell populations being 50% each. Create the gates to detect megakaryocyte population by selecting the “treatment keyword” (Figure 4A).
  h. Next, drop gates CD45 vs CD235 for red cell population and CD45 vs CD41/CD61 for immature megakaryocyte population. Using the double positive population for megakaryocytes, extend the gate for CD42b^+^ population demonstrating mature megakaryocytes.
  i. Complete step 6g. for all samples in the workspace by a simple “copy” -> “paste” function.
  j. To generate a tSNE plot, select the concatenated file, click on the “workspace” tab and choose tSNE plugin. A pop-up window appears which allows you to select the fluorophores to include in the analysis.
  k. Select the following iterations as shown in Figure 4B. Select “Run”.
    ***Note:*** For the purpose of analysis in this protocol, we selected CD45 (APC-Vio 770), CD41/CD61 (FITC), CD235 (PE) and CD42 (APC). Iterations can be adjusted depending on how separated one expects the clusters to be formed. In our opinion, upon testing Iterations = 500, did not demonstrate a change. Therefore, we use 1000 iterations each time we run the tSNE plot plugin. If this plugin is unavailable in the FlowJo version being used, it can be downloaded from the following website: https://www.flowjo.com/exchange.
  l. To edit the tSNE plot, open the layout editor by clicking this symbol . Drag and drop the concatenated file into the editor. Double click on the plot, a pop-up window appears.
  m. Select tSNE_1 on the x-axis and tSNE_2 on the y-axis. Click “OK”. The plot appears as shown in Figure 4C.
  n. Right click on the plot, choose “Make multigraph overlays” and select the “Multigraph Color Mapping” option. This generates a plot as also seen in Figure 4D.
  o. To visualize the results duplicate plot 4C twice. Drag and drop “1” generated in Bullet point 6f to one of the plots and similarly “2” to another plot.
  p. Finally, Select the colors by clicking on the color boxes in Figure 4E.
  q. The results are now ready to interpret. Figure 4F demonstrates that upon treatment, the mature megakaryocyte population (dark blue) is implicated when compared to the untreated sample.
    ***Note:*** The plot 4C can be duplicated equal to the number of samples being analyzed.
7. MACS isolation for megakaryocyte population.
  ***Note:*** Progenitors and megakaryocytes on Day 14 are harvested and subjected to magnetic-activated cell sorting (MACS selection) with CD61 microbeads.
  a. After harvest pass cells through a 100 μm cell strainer into a 50 mL Falcon tube.
  b. Wash the strainer with 10 mL PBS at room temperature (∼18°C) and collect cells by centrifugation at 400 g for 5 minutes.
  c. Resuspend cell pellet in 10 mL PBS and centrifuge at 400 g for 5 minutes.
  d. Fill falcon tube with 10 mL ice cold MACS buffer.
  e. Subject samples to MACS selection with CD61 microbeads as per instructions of the manufacturer.
  f. Collect CD61+ cells and perform cell counting.
  g. Continue with further functional assays of choice, such as co-culture with other cell types, RNA isolation, ELISA and flow cytometry analysis.^1^
    ***Note:*** Further characterization of megakaryocytes is conducted by Romanowski staining to reveal megakaryocyte morphology with budding and proplatelet formation and by transmission electron microscopy (TEM) to show megakaryocyte features, such as the demarcation membrane system (DMS) and granules, as described.^1^

## Expected outcomes

Patient iPS cells represent unique opportunities for disease modelling and compound screening, since the disease specific and/or associated mutations are preserved in iPS cells and the progeny derived thereof, including megakaryocytes. This entails the risk that the mutational load affects iPS cell differentiation output. The protocol described here has proven to yield efficient and robust megakaryocyte differentiation for a large panel of patient iPS cell lines containing an array of known mutations and mutations of unknown significance.^2^^,^^3^ Mutations and single nucleotide polymorphisms (SNP) include ABL1, ASXL1, CBL, CALR, EZH2, JAK2, KIT, SETBP1, MPL, NFE2, NRAS, PDGFRA, RUNX1, SRSF2, TP53 and TET2. Thus, the protocol is particularly suited to disease modelling and compound screening.

Frequently, human iPS cell differentiation into megakaryocytes and platelets aims at maximizing cell production for cell therapy using a high producer iPS cell line.^8^^,^^9^ The protocol described here aims at differentiation of a large array of individual patient iPS cells for disease modeling and compound screening to allow (i) disease prediction and (ii) advice on the medication, which is tailored to the patient’s need.

We integrate in the workflow functional readouts, such as the bioluminescence CellTiter-Glo® assay and flow cytometry analysis with megakaryocyte signature antibodies. CellTiter-Glo® assay assesses cell viability upon compound treatment. Flow cytometry and tSNE based visualization of data assesses megakaryocyte markers or its derivation due to mutations and compound treatment. Additionally, it also allows lineage tracing and quantification of changes in marker expression throughout the differentiation process. Further functional assays can be readily integrated into the workflow.

## Quantification and statistical analysis

All experiments were performed in at least three independent biological replicates. Flow cytometry data were analyzed using FlowJo v10.10 with consistent gating strategies.

## Limitations

The generation of iPS cells from patient samples might be hampered by the disease-causing driver mutations and/or disease associated mutation, age and the genomic profile of the patient. In addition, the mutational load might also affect iPS cell differentiation towards megakaryocytes. To overcome these limitations, we routinely derive multiple iPS cell lines from individual patients, which are then assessed for pluripotency, growth and differentiation potential.^1^^,^^3^ Two to three representative iPS cell lines per patient are then selected for further studies.

## Troubleshooting

### Problem 1

Inefficient EB formation.

### Potential solution

Efficient EB formation is critical for high progenitor output. Inefficient EB formation can be due to multiple reasons, most prominently due to poor iPS cell quality. High passage numbers, e.g., of CRISPR edited iPS cell clones, might also have a negative impact on EB formation. Employ only high-quality hiPS cell lines at low passage numbers for EB formation.

### Problem 2

Frequently, iPS cells of feeder free cultures tend to be less efficient to aggregate into EB.

### Potential solution

Maintaining iPS cells on MEF feeder^10^ for 2–3 passages prior to EB formation helps for efficient EB formation.

### Problem 3

Low starting cell density and/or high cell death during single cell seeding impact EB formation.

### Potential solution

**Figure 5:**
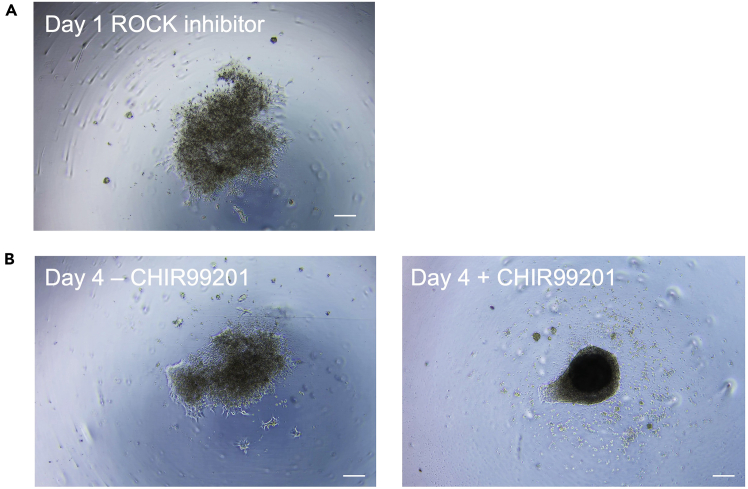
Problem 3- Optimization of EB formation (A) EB formation on Day 1 with ROCK inhibitor shows a weak core. (B) EB formation in the presence of CEPT cocktail between Day 0 and 2 with subsequent addition of CHIR99201 on Day 2 helps to form a compact core. Scale bar depicts 400 μm.

Cell numbers seeded per well of 96-well plate might need to be optimized for each iPS cell line and 3000–4000 cells per well is a good starting point. Switching from ROCK inhibitor (Figure 5A) to CEPT cocktail improved EB formation (Figures 2B and 2C). Similarly, supplementation with CHIR99201 to activate WNT signaling and mesoderm commitment yields more compact core formation (Figure 5B +CHIR).

### Problem 4

**Figure 6:**
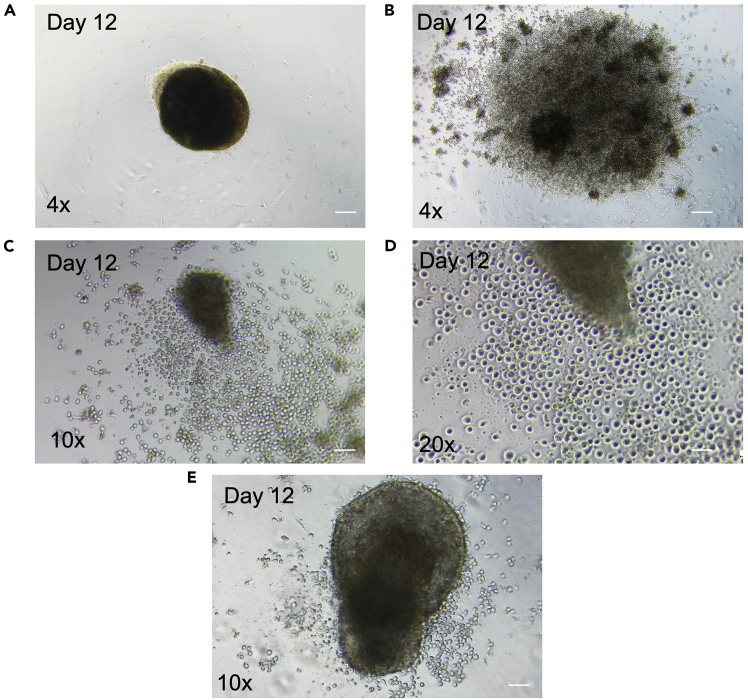
Problem 4- EB morphology and progenitor output (A) Compact EB on Day 12. (B–E) EB devoid of the perfect spherical shape producing progenitors. Scale bar is 400, 160, 80 μm for 4x, 10x and 20x magnification, respectively.

Compact EB with smooth border and dark center might not produce progenitors (Figure 6A).

### Potential solution

Key to effective hematopoietic progenitor production by EB formation is to confirm smooth borders and spherical structures of EB. However, EB with a rough periphery (Figures 6B–6E) and disintegrated core might also produce progenitors. Therefore, please consider continuing the experiments irrespective of EB shape.

### Problem 5

Antibiotics impact mesoderm commitment and thus are not included in the initial days of EB culture (Day 0–8), which entails the risk of contamination.

### Potential solution

In the event of contamination plates need to be removed from the incubator and discarded. If the contamination takes place in only few individual wells after Day 8, Penicillin/Streptomycin or any other antibiotic should be added to the medium. Penicillin/Streptomycin and antibiotics may have an impact on progenitor formation but only a minor effect on progenitor expansion.

## Resource availability

### Lead contact

Requests for further information and resources should be directed to and will be fulfilled by the lead contact, Martin Zenke (martin.zenke@rwth-aachen.de).

### Technical contact

Technical questions on executing this protocol should be directed to and will be answered by the technical contact, Payal Chawla (pchawla@ukaachen.de).

### Materials availability

This study did not generate new unique reagents.

### Data and code availability

Any additional information required to reanalyze the data reported in this paper is available from the lead contact upon request.

## Acknowledgments

The graphical abstract and Figure 2A were created in https://BioRender.com. This work was funded by 10.13039/501100001659German Research Foundation (Deutsche Forschungsgemeinschaft; DFG) to M.Z. (428858617) as part of the Clinical Research Unit (CRU344). M.A.S.d.T. was funded by a CAPES-Alexander von Humboldt postdoctoral fellowship (99999.001703/2014-05). M.A.S.d.T. and P.C. are funded by a donation from U. Lehmann.

## Author contributions

Methodology, M.A.S.d.T., N.F., P.C., and M.Z.; validation and formal analysis, P.C.; investigation, P.C.; resources, M.Z.; visualization, P.C. and M.Z.; writing, P.C. and M.Z.; reviewing and editing, M.A.S.d.T., N.F., P.C., and M.Z.

## Declaration of interests

The authors declare no competing interests.
